# Development and validation of a diagnostic model for cerebral small vessel disease among rural older adults in China

**DOI:** 10.3389/fneur.2024.1388653

**Published:** 2024-07-05

**Authors:** Chunyan Li, Jiafeng Wang, Xiaodong Han, Yuanjing Li, Keke Liu, Mingqing Zhao, Tao Gong, Tingting Hou, Yongxiang Wang, Lin Cong, Lin Song, Yifeng Du

**Affiliations:** ^1^Key Laboratory of Endocrine Glucose & Lipids Metabolism and Brain Aging, Ministry of Education, Department of Neurology, Shandong Provincial Hospital Affiliated to Shandong First Medical University, Jinan, Shandong, China; ^2^Department of Neurology, Shandong Provincial Hospital, Shandong University, Jinan, Shandong, China; ^3^Aging Research Center and Center for Alzheimer Research, Department of Neurobiology, Care Sciences and Society, Karolinska Institutet-Stockholm University, Stockholm, Sweden; ^4^Shandong Provincial Clinical Research Center for Neurological Diseases, Jinan, Shandong, China

**Keywords:** cerebral small vessel disease, diagnostic model, blood pressure, inflammation, population-based study

## Abstract

**Objectives:**

Cerebral small vessel disease (CSVD) visible on MRI can be asymptomatic. We sought to develop and validate a model for detecting CSVD in rural older adults.

**Methods:**

This study included 1,192 participants in the MRI sub-study within the Multidomain Interventions to Delay Dementia and Disability in Rural China. Total sample was randomly divided into training set and validation set. MRI markers of CSVD were assessed following the international criteria, and total CSVD burden was assessed on a scale from 0 to 4. Logistic regression analyses were used to screen risk factors and develop the diagnostic model. A nomogram was used to visualize the model. Model performance was assessed using the area under the receiver-operating characteristic curve (AUC), calibration plot, and decision curve analysis.

**Results:**

The model included age, high blood pressure, white blood cell count, neutrophil-to-lymphocyte ratio (NLR), and history of cerebral infarction. The AUC was 0.71 (95% CI, 0.67–0.76) in the training set and 0.69 (95% CI, 0.63–0.76) in the validation set. The model showed high coherence between predicted and observed probabilities in both the training and validation sets. The model had higher net benefits than the strategy assuming all participants either at high risk or low risk of CSVD for probability thresholds ranging 50–90% in the training set, and 65–98% in the validation set.

**Conclusion:**

A model that integrates routine clinical factors could detect CSVD in older adults, with good discrimination and calibration. The model has implication for clinical decision-making.

## Introduction

Cerebral small vessel disease (CSVD) refers to as various pathologic processes affecting the small arteries, arterioles, capillaries, and probably venules of the brain (1). The MRI markers for CSVD manifest as recent small subcortical infarcts, brain atrophy, white matter hyperintensity (WMH), lacunes, microbleeds, and enlarged perivascular space (EPVS) (2). As people age, CSVD is increasingly common. For instance, The prevalence of WMH affecting approximately 5% of individuals aged 50 years and to almost 100% of individuals aged 90 years (3). Similarly, the prevalence of CMB rises from 6.5% among individuals aged 45–50 years to 35.7% among those of 80 years and older (4).

In addition to older age, previous studies have suggested that CSVD is associated with conventional vascular risk factors such as smoking, hypertension, diabetes mellitus, dyslipidemia, and obesity (5, 6). Furthermore, a community-based study showed that CSVD was associated with higher white blood cell (WBC) count, neutrophil count (NC), neutrophil-to-lymphocyte ratio (NLR), and systemic immune-inflammation index (SII) (7). This supports the view that systemic inflammation is involved in the pathogenesis of CSVD (8).

CSVD has been associated with series of clinical sequelae, such as clinical stroke, cognitive impairment, and gait and balance disturbances (9). It’s reported that CSVD contributes to 25% of ischaemic strokes and most hemorrhagic strokes (9). Furthermore, CSVD is the prime cause of vascular dementia, and contributes to up to 45% of all dementia (10). Despite the substantial impact of CSVD on health, there has no available effective treatment. Thus, early detection of CSVD may provide the potential for preventive or therapeutic interventions to delay or prevent its clinical sequelae because most risk factors for CSVD are modifiable or clinically manageable. A large-scale population-based study of middle-aged people found that adding NC to the basic model of traditional vascular risk factors could significantly improve the accuracy of detecting CSVD (7). However, a simple, practical, and clinically useful model for detecting CSVD among older adults remains to be developed.

In this population-based study of older adults who were living in rural communities, we sought to (1) explore the possible risk factors associated with CSVD; (2) develop and validate a diagnostic model to detect CSVD; and (3) evaluate the clinical net benefits of the model.

## Materials and methods

### Study design and participants

The protocols of MIND-China and MRI sub-study were reviewed and approved by the Ethics Committee at Shandong Provincial Hospital in Jinan, Shandong, China. Written informed consent was obtained from all participants or proxies.

We used data derived from the baseline assessments of the Multimodal INtervention to delay Dementia and disability in the rural China (MIND-China) that is a participating project in the World-Wide FINGERS Network, as described previously (11, 12). Briefly, the MIND-China study targeted people aged 60 years and older and living in 52 rural communities of Yanlou Town, Yanggu County, western Shandong province, China. From March to September 2018, 5,765 participants were examined for MIND-China. Of them, 1,304 participants from 26 villages randomly selected from all the 52 villages accomplished the structural brain MRI scans.

### MRI acquisition and processing

All participants underwent the brain MRI scans either on the Philips Ingenia 3.0 T MR System (Philips Healthcare, Best, The Netherlands) in Southwestern Lu Hospital or on the Philips Achieva 3.0 T MR System (Philips Healthcare, Best, The Netherlands) in Liaocheng People’s Hospital. The MRI sequences included the sagittal 3D sT1-weighted, axial T2-weighted, sagittal 3D Fluid-attenuated inversion recovery (FLAIR) images, and axial susceptibility weighted imaging (SWI). The detailed parameters of the core MRI sequences have been reported previously (13).

We assessed the following four MRI markers for CSVD. Cerebral microbleeds (CMBs) were focal, rounded hypodense lesions measuring less than 5 mm in diameter on SWI. The CMBs was quantitatively acquired by AccuBrain® (BrainNow Medical Technology Ltd., Shenzhen, Guangdong, China) as described previously (14, 15). Briefly, CMBs were detected on SWI images via a fully connected network that was trained by deep learning technique. For a given SWI image, the network showed CMB location by exporting a probability map.

The visual assessment of lacunes, WMH, and PVS was performed by two well-trained raters (M.Z. for EPVS and J.W. for lacunes and WMH) according to the standards for reporting vascular changes on neuroimaging 1 (STRIVE-1) (2). The raters were blinded to participants’ clinical data and under the supervision of an experienced clinical neurologist (L.S) and an experienced neuroradiologist (T.G.). Lacunes were rounded or ovoid lesions, 3–15 mm in diameter, generally in the intensity of cerebrospinal fluid (CSF) signal on T2 and FLAIR sequences. The trained rater (J.W.) counted lacunes in each hemisphere on FLAIR sequence, and then added up the numbers of lacunes in bilateral hemispheres. WMH was defined as symmetrical hyperintense on T2 images in the brain white matter, and was evaluated in periventricular and deep white matter region according to the Fazekas scale (16). We defined periventricular WMH (PWMH) and deep WMH (DWMH) following the “continuity to ventricle” rule (17). The rater (J.W.) evaluated WMH on the slice with the most severe white matter lesions. We defined EPVS as small (<3 mm) punctate (if perpendicular) or linear (if longitudinal to the plane of scan) hyperintensities on T2 images. We rated EPVS using the validated semiquantitative scale (18). Briefly, the rater (M.Z.) visually counted bilateral EPVS in basal ganglia (BG) and centrum semiovale (CSO) on all slice, and categorized EPVS in BG and CSO according to the highest counts on the slice and hemisphere with the most EPVS.

Six months after the initial assessment, the rater re-assessed MR images of 200 randomly selected subjects for lacunes and WMH, which yielded the weighted ĸ statistic of 0.84 for lacunes, 0.86 for DWMH, and 0.89 for PWMH. Similarly, three months after the initial assessment, the rater re-evaluated EPVS in MRI images of 30 randomly selected subjects, which yielded a weighted ĸ statistic of 0.75 for BG-EPVS and 0.74 for CSO-EPVS.

Total CSVD score was assessed as previously reported (19). One point was assigned for the presence of: (a) lacunes; (b) CMBs; (c) moderate-to-severe EPVS (>10) in BG; and (d) DWMH (Fazekas score 2–3) or PWMH (Fazekas score 3). We dichotomized the total CSVD score into the absence of CSVD (total CSVD score = 0) and the presence of CSVD (total CSVD score ≥ 1) (7).

### Data collection and definitions of candidate risk factors

In March–September 2018, extensive data were collected by trained staff through face-to-face interviews, clinical examinations or laboratory tests. According to the literature (5–8, 10, 20), we selected the following candidate risk factors for CSVD: age, sex, education, body mass index (BMI), smoking, alcohol drinking, high blood pressure, pulse pressure, history of cerebral infarction, fasting blood glucose (FBG), low-density lipoprotein (LDL), WBC count, NC, NLR, and SII. Education was dichotomized into low education (primary school and below) and high education (middle school and above). Alcohol consumption and smoking status were categorized as current, former, and never drinking or smoking, respectively. Arterial pressure was measured as described previously (21), and high blood pressure was defined as systolic pressure ≥ 140 mmHg or diastolic pressure ≥ 90 mmHg. FBG and LDL was measured using an automatic biochemical analyzer (DIRUICS-600B; DIRUI Corporation, Changchun, China) (22). The automated blood cell analyzer (BC1800, Mindray Corporation, Shenzhen, China) was used for routine blood tests (12). NLR (neutrophil count/lymphocyte count) and SII (platelet count × neutrophil count/lymphocyte count) were calculated based on the absolute NC (×10^9^/L), lymphocyte count (×10^9^/L), and platelet count (×10^9^/L).

We defined clinical risk factors (i.e., high blood pressure, high pulse pressure, obesity, and high FBG) according to current clinical criteria, as described above. For laboratory measurements (e.g., LDL, WBC, NC, NLR, and SII), abnormal values were defined according to the optimal cutoff points determined using the receiver operating characteristic (ROC) analyses and Youden index (Supplementary Table S1).

### Statistical analysis

We performed the analyses among participants with complete data. We presented frequencies (%) for categorical variables and the median (interquartile range, IQR) for continuous variables. The Chi-square test or Mann–Whitney test was used for the comparisons of categorical or continuous variables, respectively. Univariate logistic regression analyses were performed to screen potential risk factors at the level of *p* < 0.10, and multivariable logistic regression analyses using backward stepwise approach was used to select independent risk factors associated with CSVD.

We assessed the model performance using discrimination and calibration. The discrimination refers to as the model’s ability to distinguish between high- and low-risk participants and was assessed by calculating the area under the ROC (AUC) that ranged from 0.5 (no better than chance) to 1.0 (perfect discrimination) (23). The calibration was defined as the agreement between the predicted and observed probability. It was determined by Hosmer-Lemeshow test and calibration curve (23), where people were evenly divided into ten groups based on decile of predicted risk, and the predicted probability was plotted against the observed probability. A diagonal line with intercept of 0 and slope of 1 represented ideal calibration.

We assessed the clinical usefulness of the model using decision curve analysis (DCA), which compared the net benefit of using the diagnostic model vs. the strategy of assuming that all people were at high or low risk of CSVD (24). Graphically, the line parallel to the x-axis was drawn to show no net benefit when assuming all people with low CSVD risk and no intervention given, whereas the solid black curve represented all people with high CSVD risk and received intervention. The DCA curve (black dotted curve) was drawn for the established diagnostic model, and the curve with the highest net benefit corresponded to a higher clinical value.

All analyses were performed using IBM SPSS Statistics for Windows, Version 26.0 (IBM Corp., Armonk, NY, USA) and Stata Statistical Software: Release 15.0 (Stata Corp LLC., College Station, TX, USA) for Windows. Two-sided *p* < 0.05 was considered statistically significant.

## Results

### Characteristics of study participants

**Figure 1:**
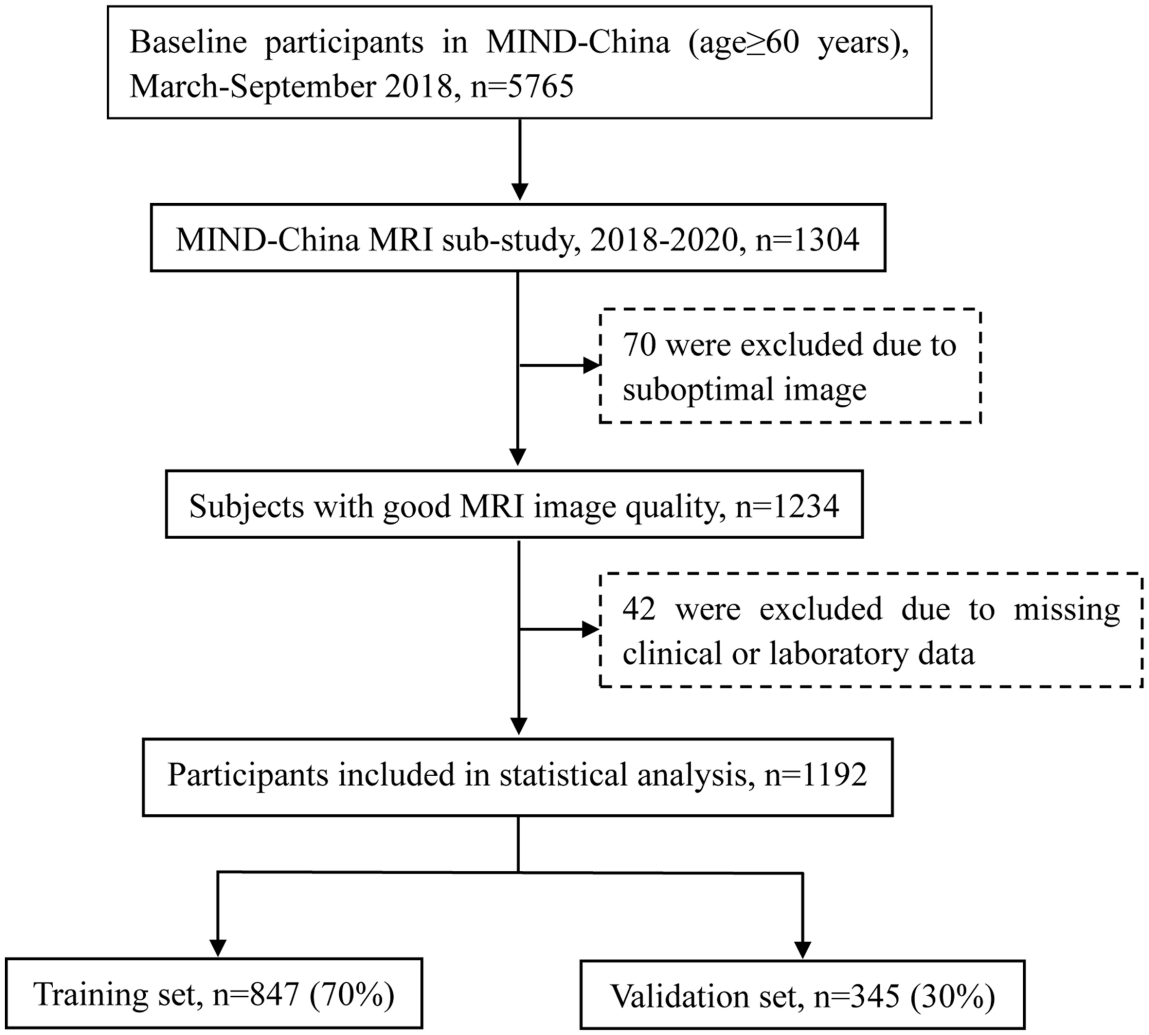
Flowchart of the study participants. MRI, magnetic resonance imaging; MIND-China, Multimodal Interventions to Delay Dementia and Disability in Rural.

In 1304 participants accomplished the structural brain MRI scans, we excluded 112 participants due to suboptimal image quality (*n* = 70) or missing data on clinical features and laboratory measures (*n* = 42), leaving 1,192 participants for the current analysis. These participants were randomly divided into the training set (70%, *n* = 847) and the validation set (30%, *n* = 345). Figure 1 shows the flowchart of the study participants.

The median age of the 1,192 participants was 69 years (IQR: 66–72 years), 58.56% were female, and 80.37% had limited education (i.e., primary school or no school education) (Table 1). Out of these, 934 (78.36%) were presented with CSVD (i.e., total CSVD score ≥ 1), including 659 (77.80%) in the training set and 275 (79.71%) in the validation set. There was no statistical significance between the two datasets with respect to all the examined variables (Table 1).

**Table 1:** Characteristics of participants in the training and validation sets.

Characteristics	Total sample (*n* = 1,192)	Training set (*n* = 847)	Validation set (*n* = 345)	*p* value
Demographic factors				
Age (years)	69 (66, 72)	69 (66, 73)	69 (66, 72)	0.95
Female, *n* (%)	698 (58.56)	495 (58.44)	203 (58.84)	0.90
Educational level, *n* (%)				0.91
Low (primary school or below)	958 (80.37)	680 (80.28)	278 (80.58)	
High (middle school or above)	234 (19.63)	167 (19.72)	67 (19.42)	
Risk factors				
Body mass index (kg/m^2^)	24.90 (22.50, 27.10)	24.80 (22.70, 27.10)	24.90 (22.10, 27.35)	0.72
Current smoking, *n* (%)	242 (20.30)	169 (19.95)	73 (21.16)	0.64
Current drinking, *n* (%)	379 (31.80)	270 (31.88)	109 (31.59)	0.92
Systolic pressure (mmHg)	142 (130, 156)	142 (130, 156)	141 (130, 158)	0.97
Diastolic pressure (mmHg)	85 (79, 92)	85 (79, 92)	84 (79, 91)	0.24
Pulse pressure (mmHg)	56 (46, 67)	56 (46, 67)	58 (46, 68)	0.38
Cerebral infarction, *n* (%)	144 (12.08)	106 (12.51)	38 (11.01)	0.47
Laboratory data				
FBG (mmol/L)	5.24 (4.89, 5.78)	5.23 (4.91, 5.78)	5.27 (4.84, 5.77)	0.79
LDL (mmol/L)	2.67 (2.31, 3.01)	2.66 (2.29, 3.00)	2.70 (2.33, 3.04)	0.40
white blood cell count (×10^9^/L)	5.60 (4.90, 6.70)	5.60 (4.80, 6.70)	5.70 (4.90, 6.60)	0.63
Neutrophil count (×10^9^/L)	3.40 (2.80, 4.20)	3.50 (2.80, 4.20)	3.40 (2.90, 4.20)	0.84
NLR	2.00 (1.59, 2.55)	2.00 (1.59, 2.56)	2.00 (1.59, 2.52)	0.85
SII (×10^9^/L)	402.00 (303.13, 529.98)	402.32 (302.64, 530.06)	401.75 (307.54, 527.85)	0.80

Data were median (IQR), unless otherwise specified. FBG, fasting blood glucose; LDL, low-density lipoprotein; NLR, neutrophil-to-lymphocyte ratio; SII, systemic immune-inflammation index; IQR, interquartile range.

### Independent risk factors for CSVD in the training set

Univariate logistic regression analysis suggested nine of the 15 candidate risk factors were associated with CSVD at the *p* < 0.10 (Table 2). These nine risk factors were then entered into the multivariable logistic regression model, and five risk factors, i.e., older age, high blood pressure, history of cerebral infarction, increased WBC count, and increased NLR, were independently associated with CSVD and selected for the final diagnostic model (Table 2).

**Table 2:** Univariate and multivariate analysis of risk factors for CSVD in training set.

Variables	Univariate analysis		Multivariate analysis	
	OR (95% CI)	*p* value	OR (95% CI)	*p* value
Age groups (years)	-	<0.001	-	<0.001
60–64	1.00 (reference)	-	1.00 (reference)	-
65–69	1.42 (0.90, 2.26)	0.133	1.49 (0.92, 2.41)	0.108
70–74	2.56 (1.55, 4.22)	<0.001	2.82 (1.67, 4.76)	<0.001
≥75	4.51 (2.21, 9.22)	<0.001	4.32 (2.07, 9.03)	<0.001
Sex (female vs. male)	0.82 (0.59, 1.14)	0.232	-	-
Educational level (high vs. low)	1.32 (0.86, 2.02)	0.208	-	-
Obesity (BMI ≥28 kg/m^2^)	1.52 (0. 97, 2.39)	0.067	-	-
Current smoking	1.12 (0.74, 1.69)	0.603	-	-
Current alcohol drinking	1.06 (0.75, 1.51)	0.732	-	-
High blood pressure	2.57 (1.85, 3.59)	<0.001	2.47 (1.75, 3.48)	<0.001
High pulse pressure (≥60 mmHg)	1.97 (1.39, 2.77)	<0.001	-	-
History of cerebral infarction	2.02 (1.21, 3.63)	0.019	1.84 (1.00, 3.38)	0.052
High FBG (≥7.0 mmol/L)	1.50 (0.81, 2.78)	0.200	-	-
High LDL (≥2.5 mmol/L)	1.14 (0.82, 1.59)	0.446	-	-
High white blood cell count (≥7.0 × 10^9^/L)	2.36 (1.45, 3.85)	0.001	2.10 (1.26, 3.51)	0.005
High neutrophil count (≥3.6 × 10^9^/L)	1.60 (1.15, 2.23)	0.006	-	-
High NLR (≥2.1)	1.72 (1.23,2.40)	0.001	1.77 (1.24, 2.52)	0.002
High SII (≥413×109/L)	1.66 (1.19, 2.32)	0.003	-	-

CSVD, cerebral small vessel disease; BMI, body mass index; FBG, fasting blood glucose; LDL, low-density lipoprotein; NLR, neutrophil-to-lymphocyte ratio; SII, systemic immune-inflammation index; OR, odds ratio; CI, confidence interval.

### Development of a nomogram in the training set

**Figure 2:**
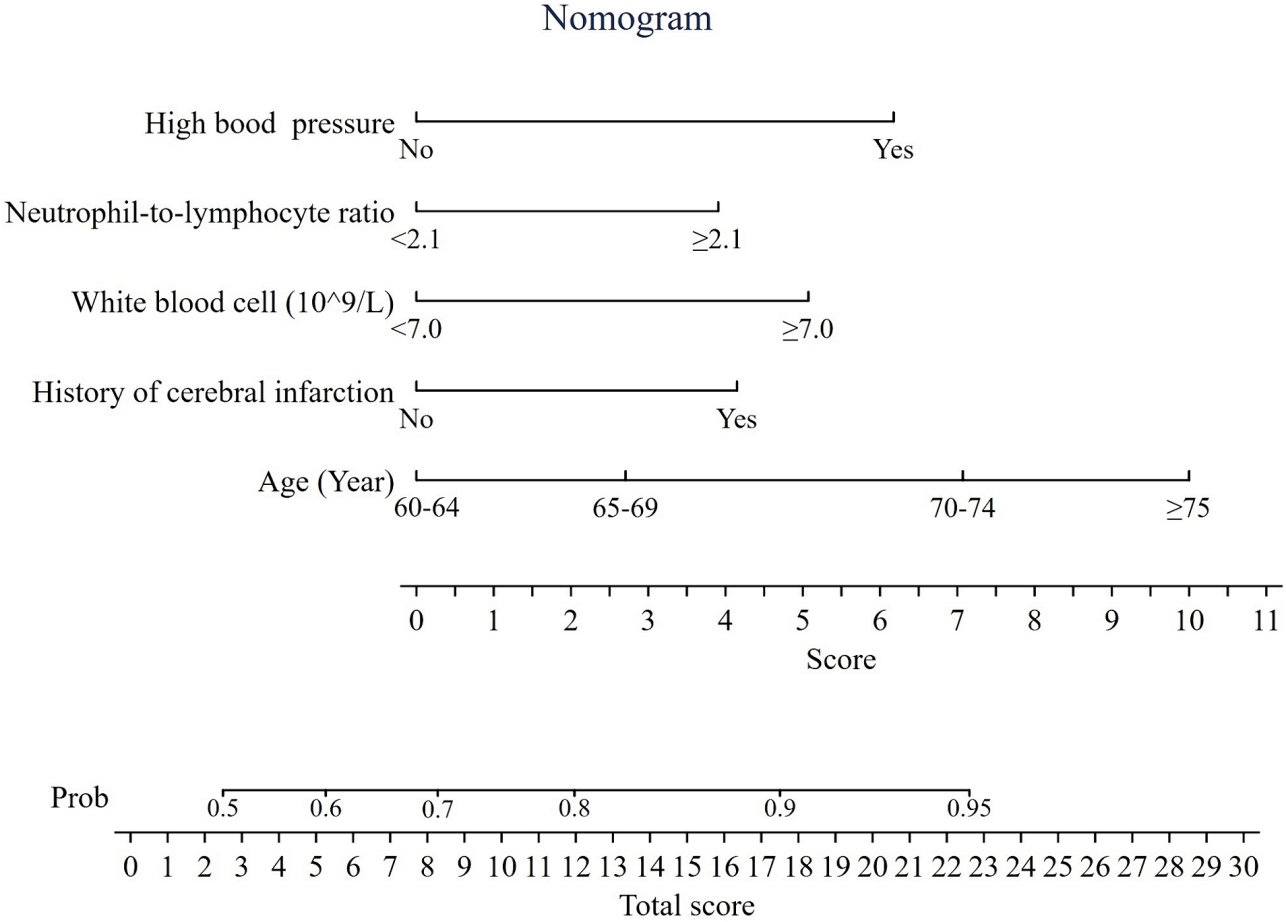
Nomogram predicting the probability of having cerebral small vessel disease. Total score = 3.9 (if NLR ≥2.1) +5.1 (if WBC ≥ 7.0 × 109/L) + 6.2 (if with high blood pressure) +4.2 (if with a history of cerebral infarction) + 0/2.7/7.1/10 (if age range of 60–64, 65–69, 70–74, and ≥ 75 years, respectively).

A nomogram was built based on the multivariable logistic regression (Figure 2). Each variable was assigned a weighted score based on odds ratio. A total score was generated by adding each weighted score of the risk factors, then the probability of having CSVD was determined by projecting the total score to the total point scale. For example, a person aged 62 years with a history of cerebral infarction, high blood pressure, WBC count of 7.0 × 10^9^/L, and NLR of 2.2 had a total point 19.4, representing approximately 92% probability of having CSVD (Supplementary Figure S1).

### Internal validation of the diagnostic model

**Figure 3:**
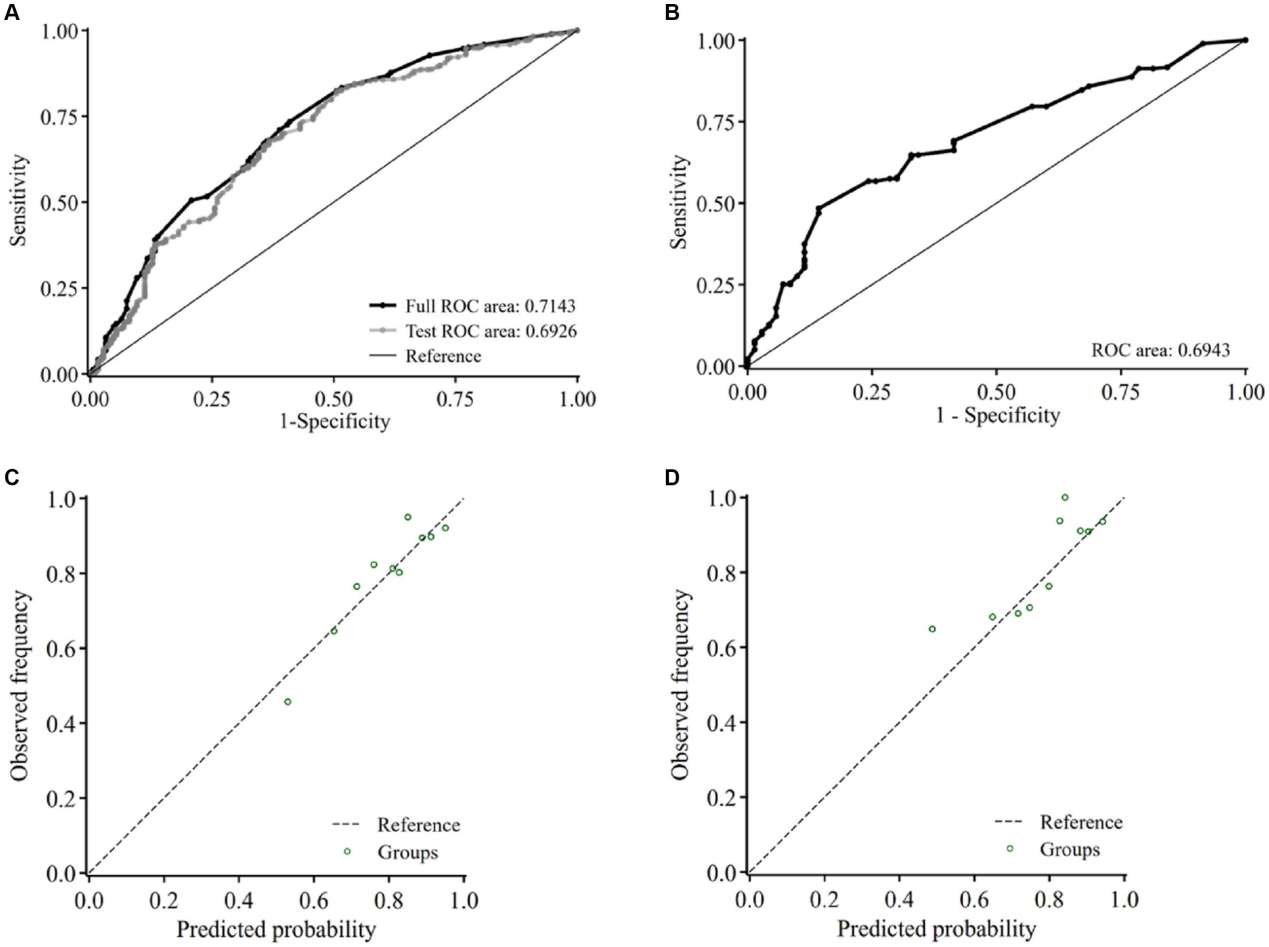
Internal validation of the model in the training set and validation set. Receiver-operating characteristic curve and the corresponding area of the diagnostic model before (black) and after 10-fold cross-validation (gray) in the training set **(A)** and the validation set **(B)**. Calibration curve of the model in the training set **(C)** and the validation set **(D)** of the presence of CSVD. Abbreviations: ROC, receiver operating characteristic curve.

The diagnostic model yielded AUC of 0.71 (95% CI, 0.67–0.76) in the training set (Figure 3A) and 0.69 (95% CI, 0.63–0.76) in the validation set (Figure 3B). Ten-fold cross-validation was performed in the training set, with the AUC being 0.69 (Figure 3A). The calibration plot showed a good agreement between the predicted and the observed probabilities of having CSVD in both the training (Figure 3C) and the validation sets (Figure 3D), which was verified by Hosmer-Lemeshow test, with χ^2^ being 10.50 (*p* = 0.40) and 10.36 (*p* = 0.41) for the training and the validation set, respectively, indicating no significant difference between the predicted and observed probabilities.

### Decision curve analysis

**Figure 4:**
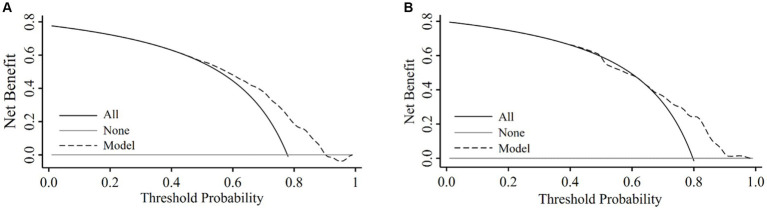
Decision curve obtained from plotting the net benefit of detecting CSVD in the training set and validation set. Decision curve analysis in the training set **(A)** and the validation set **(B)**.

We assessed the clinical utility of the nomogram using DCA. In the training set (Figure 4A), the curve of applying the model crossed the curves of treating all participants as having low and high risk approximately at threshold probability of 90 and 50%, respectively, suggesting that the diagnostic model had higher net benefits for risk thresholds between 50 and 90%. In the validation set (Figure 4B), the model had higher net benefits than the strategy assuming all participants either at high risk or low risk of CSVD for risk thresholds between 65 and 98%.

## Discussion

Using data from a population of rural-dwelling older adults, we developed and validated a diagnostic model for detecting CSVD, and the model included age, high blood pressure, history of cerebral infarction, WBC count, and NLR. The AUC and calibration plot confirmed the model’s relatively good discriminative ability and calibration. DCA metrics indicated the model had good clinical application value.

Our diagnostic model yielded AUC of 0.71 in the training set and 0.69 in the validation set, which indicated an acceptable discriminative ability. It has been reported that AUC values higher than 0.7 are generally considered fair and values higher than 0.9 excellent (25). To our best knowledge, little diagnostic models have been developed for CSVD. Only a hospital-based study reported a predictive model incorporating nine predictors, of which AUC of the model was 0.85 (26). Though higher than ours, most variables incorporated in the model were unconventional indicators, which may limit the generalizability of the model, especially for rural-dwelling older adults. In addition, we use Hosmer-Lemeshow test to assess the model’s calibration. It has been reported that Hosmer-Lemeshow test is susceptible to sample size, and with large sample sizes, a minor difference between the predicted and the observed risk could be identified to be statistically significant (i.e., false-positive results) (27). Furthermore, Hosmer-Lemeshow test is powerless in detecting overfitting of predictor effects (24). However, calibration plot, another way to assess calibration which is preferred over the Hosmer-Lemeshow tests (24), confirmed a high coherence between predicted and observed probabilities in our study.

In this study, older age was the strongest contributing factor to our nomogram, consistent with previous studies (5, 6). High blood pressure was an independent risk factor for CSVD in the model. Long-term high blood pressure could cause endothelial dysfunction and subsequent blood–brain barrier (BBB) dysfunction that was a driving force leading to CSVD. Increased BBB permeability allowed the leakage of fluid and blood products into the perivascular spaces, leading to EPVS (28). Moreover, chronic hypertension can cause structural alterations to cerebral small vessels (e.g., thickening of the vessel walls and narrowing of the lumen), thus, leading to chronic hypoperfusion and cerebral ischemic lesions (29).

We identified two indicators of systemic inflammation, WBC count and NLR, that contributed to the diagnostic model for CSVD. Increased WBC count, a risk factor for arteriosclerosis, has been associated with coronary heart disease and stroke (30, 31). However, very few population-based studies have investigated the association between WBC and CSVD. A community-based study showed middle-aged people with CSVD had higher WBC count (7). A Mendelian randomization study revealed that higher WBC count was associated with small vessel stroke (32). NLR has been a strong predictor of stroke and cardiovascular disease (33, 34), but the relationship between NLR and CSVD remains poorly understood. Previously, CSVD was found to be associated with a higher NLR in one population-based study of middle-aged adults (7), but not in another community-based study and a hospital-based study of middle-aged and older adults (35, 36). The discrepant findings across studies may be partly attributed to different characteristics of study participants (e.g., age, education, and settings).

The following potential mechanisms may underlie the associations of WBC and NLR with CSVD. Firstly, increased WBC could adhere to vascular endothelium, resulting in endothelial dysfunction and subsequent atherosclerosis and BBB damage (7). Secondly, a higher NLR indicates increased neutrophils or decreased lymphocytes or both. High neutrophils could release various inflammatory cytokines, triggering inflammatory cascades (37). In contrast, lymphocytes could be a healing promotor by secreting interleukin-10 (37).

The major strength of our study was the relatively large-scale sample that engaged rural-dwelling older adults in western Shandong Province, China, a sociodemographic group that has been rarely targeted in brain aging research. Our study also has limitations. Firstly, the model was developed and validated based on data from a single center, the external validation in the future would increase the generalizability of the findings. Secondly, although the four MRI markers were used in previous studies for assessing the CSVD burden (7, 19), we did not have additional MRI markers such as recent small subcortical infarcts and brain atrophy, which may underestimate the CSVD burden in older people.

As the majority of older adults with CSVD are clinically asymptomatic, early detection is crucial for effective interventions to prevent occurrence of catastrophic cerebrovascular events and cognitive consequences. Brain MRI is not cost-effective and clinically not feasible, especially in rural areas. Instead, the diagnostic model based on easily accessible variables could be simple and practical to identify CSVD at asymptomatic stage, thus assisting clinical decision-making with regard to the necessity for further MRI examination and prevention and therapeutic interventions to slow progression of CSVD and related clinical consequences. Further exploration of sensitive biomarkers for CSVD is essential, especially plasma biomarkers and genetic factors, which would help to improve model performance. Additionally, prediction model for CSVD from large scale longitudinal study to early identify those at high risk of CSVD is urgently needed.

## Conclusion

In conclusion, we developed and validated a diagnostic model by integrating five easily accessible factors for detecting CSVD in rural older adults. The model with good discrimination, calibration, and clinical benefits has the potential to detect CSVD at asymptomatic stage, and thus, provide the potential for secondary interventions of CSVD and functional consequences.

## Data availability statement

The raw data supporting the conclusions of this article will be made available by the authors, without undue reservation.

## Ethics statement

The studies involving humans were approved by the Ethics Committee at Shandong Provincial Hospital in Jinan, Shandong, China. The studies were conducted in accordance with the local legislation and institutional requirements. The participants provided their written informed consent to participate in this study.

## Author contributions

CL: Conceptualization, Validation, Formal analysis, Investigation, Methodology, Software, Writing – original draft. JW: Methodology, Validation, Writing – review & editing. XH: Methodology, Software, Writing – review & editing. YL: Methodology, Validation, Writing – review & editing. KL: Investigation, Methodology, Writing – review & editing. MZ: Investigation, Writing – review & editing. TG: Investigation, Methodology, Writing – review & editing. TH: Data curation, Resources, Writing – review & editing. YW: Data curation, Resources, Writing – review & editing. LC: Data curation, Resources, Writing – review & editing. LS: Conceptualization, Data curation, Funding acquisition, Resources, Supervision, Validation, Writing – review & editing. YD: Conceptualization, Data curation, Funding acquisition, Project administration, Resources, Supervision, Writing – review & editing.
