# Supplementary material for: Development and validation of a diagnostic model for cerebral small vessel disease among rural older adults in China
Source: Front Neurol. 2024 Jul 5;15:1388653. doi: 10.3389/fneur.2024.1388653 (PMC11258008; doi:10.3389/fneur.2024.1388653)
Supplement: Supplementary file 1 [file Data_Sheet_1.docx]

Supplementary Material

**Supplementary Table 1. The optimal cutoff points for variables determined based on Youden index.**

| **Variables** | **Youden index** | **Cutoff points** |
| --- | --- | --- |
| Low-density lipoprotein cholesterol | 0.054 | 2.4 (mmol/L) |
| White blood cell count | 0.093 | 6.9 (×10^9^/L) |
| Neutrophil count | 0.103 | 3.5 (×10^9^/L) |
| Neutrophil-to-lymphocyte ratio | 0.113 | 2.0 |
| Systemic immune-inflammation index | 0.099 | 412.0 (×10^9^/L) |


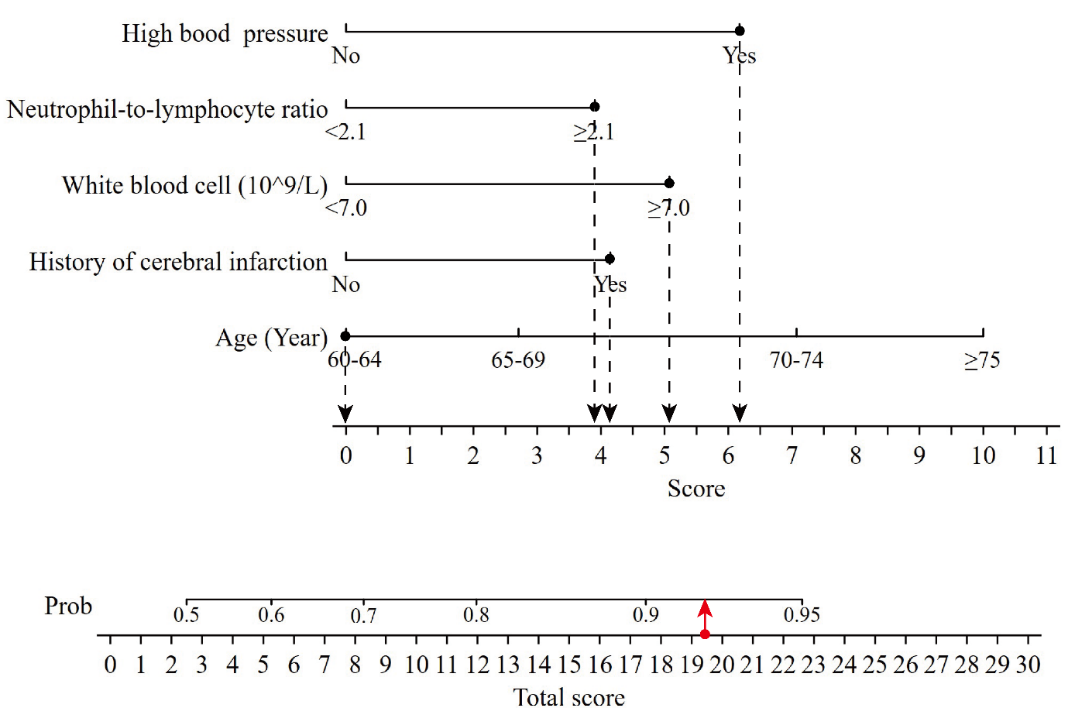


**Supplementary Figure 1. Nomogram illustration.**
